# Clinical performance of a giomer-based pit and fissure sealant with and without air-abrasion pretreatment: a 12-month randomized clinical trial

**DOI:** 10.1186/s12903-026-09691-8

**Published:** 2026-09-03

**Authors:** Yomna Sayed Khallaf, Olfat Elsayed Hassanein, Aya Mohamed Adly, Dina Kamal

**Affiliations:** 1 Conservative Dentistry Department, Faculty of Dentistry, Cairo University, 11 Al-Saraya Street, Al-Manial, Cairo, Egypt; 2 Department of Clinical Dental Sciences, Faculty of Dentistry, Ibn Sina University for Medical Sciences, Amman, 16197 Jordan

**Keywords:** Air-abrasion pretreatment, Giomer-based sealant, Pit and fissure sealant, Randomized clinical trial, Sealant retention

## Abstract

**Background:**

Pit and fissure sealants are widely used for caries prevention; however, their long-term success depends largely on retention. Giomer-based sealants containing surface pre-reacted glass ionomer fillers offer bioactive properties, yet concerns remain regarding their bonding durability when applied with mild self-etch primers. This randomized clinical trial evaluated the effect of bioactive glass air-abrasion pretreatment on the retention and caries preventive efficacy of a giomer-based sealant in young adults over 12 months.

**Methods:**

This parallel-arm randomized clinical trial included 96 participants, each contributing one eligible sound permanent molar (*n* = 48 per group). Participants were randomly allocated to either bioactive glass air-abrasion pretreatment followed by application of a giomer-based sealant (intervention group) or application of the same sealant without pretreatment (comparator group). Sealant retention and secondary caries incidence were evaluated at baseline, 6 months, and 12 months using Simonsen’s criteria, and modified United States Public Health Service (USPHS) criteria, respectively. The primary outcome was sealant retention at 12 months, whereas secondary caries incidence was assessed as a secondary outcome. Intergroup comparisons were analyzed using the Chi-square test. Intragroup comparisons were analyzed using Cochran’s Q test followed by multiple comparisons. Relative risk with 95% confidence intervals was calculated. Statistical significance was set at *p* ≤ 0.05.

**Results:**

At 6 months, complete sealant retention was observed in 91.7% of teeth in the intervention group and 75.0% in the comparator group, with no statistically significant difference between groups (*p* = 0.068). At 12 months, complete sealant retention was significantly higher in the intervention group (87.5%) than in the comparator group (33.3%) (*p* < 0.0001). Teeth in the intervention group exhibited an 81.25% lower risk of sealant retention failure compared with the comparator group (RR = 0.1875; 95% CI: 0.0864–0.4069; *p* < 0.0001). No differences in secondary caries incidence were detected between groups during the 12-month follow-up period (*p* = 1.0000).

**Conclusions:**

Bioactive glass air-abrasion pretreatment significantly improved the retention of a giomer-based fissure sealant compared with sealant application without pretreatment. No differences in secondary caries incidence were detected between groups during the 12-month follow-up period. Incorporating mechanical surface conditioning prior to sealant placement may enhance sealant retention without compromising preventive efficacy.

**Trial Registration:**

https://clinicaltrials.gov/, (NCT06003452), 15-08-2023.

## Introduction

Dental caries remains one of the most prevalent oral diseases worldwide and continues to represent a major public health challenge despite being largely preventable [1]. According to the Global Burden of Disease study, the number of individuals affected by caries in permanent teeth has increased substantially over decades and is projected to rise in the future [2].

Occlusal pits and fissures of permanent molars are particularly susceptible to caries, especially shortly after eruption, owing to their complex and plaque-retentive morphology. These anatomical features hinder effective plaque removal and promote biofilm stagnation, increasing the risk of early carious lesions. Consequently, timely protection of these surfaces using appropriate preventive strategies is critical. Recommended preventive measures include mechanical plaque control, topical fluoride application, antimicrobial agents, and pit-and-fissure sealants, all of which aim to preserve the structural integrity of newly erupted molars [3]. According to the American Dental Association guidelines, sealant application is indicated for teeth classified as ICDAS II codes 0, 1, or 2 [4, 5].

Resin-based sealants applied to acid-etched enamel currently represent the standard of care. The use of an adhesive system prior to sealant placement has been shown to enhance bond strength and retention; however, this approach involves multiple technique-sensitive steps that may prolong chairside time and reduce patient acceptance. To address these limitations, self-etching sealant systems have been introduced to simplify the clinical procedure while maintaining adequate bonding performance [6]. Among these, giomer-based sealants incorporate surface pre-reacted glass-ionomer (S-PRG) fillers, which can release multiple beneficial ions, including fluoride, sodium, aluminum, strontium, borate, and silicate. These ions contribute to acid neutralization, inhibition of demineralization, promotion of remineralization, and reduction of plaque accumulation [7]. Fluoride and strontium ions interact with hydroxyapatite to form fluoroapatite and strontium-substituted apatite, thereby enhancing enamel resistance to acid challenges. Additionally, S-PRG fillers exhibit sustained fluoride release with recharge capability, supporting their potential role in long-term caries prevention [8].

Despite these advantages, achieving durable adhesion to enamel remains challenging, particularly in newly erupted teeth. The enamel surface at this stage is often aprismatic and highly mineralized, rendering it relatively resistant to conventional acid etching [9]. This may result in insufficient micromechanical retention and increased risk of sealant failure. Consequently, enamel pre-treatment methods such as bur roughening or air-abrasion have been advocated to enhance surface conditioning; however, their clinical effectiveness remains controversial [10].

Air-abrasion using aluminum oxide particles has been widely employed for enamel surface preparation. Nevertheless, concerns persist regarding its aggressive cutting action and lack of bioactive properties. In response, bioactive glass has emerged as a promising alternative abrasive material [11]. Bioactive glass particles are composed of calcium, sodium, and phosphosilicates in proportions comparable to natural enamel hydroxyapatite, rendering them biocompatible and capable of promoting remineralization. Moreover, their slower cutting rate compared with aluminum oxide allows for a more conservative enamel preparation, potentially preserving tooth structure while improving sealant penetration and adhesion [12, 13].

Although bioactive glass air-abrasion has been investigated as an enamel-conditioning method and giomer-based sealants have demonstrated promising bioactive properties, clinical evidence evaluating the combined use of these two approaches remains limited. In particular, the effect of bioactive glass air-abrasion pretreatment on the retention and caries-preventive performance of self-etch giomer-based fissure sealants under clinical conditions has not been adequately investigated. Thus, well-designed randomized clinical trials are required to clarify the potential benefit of this combination.

Therefore, the present randomized clinical trial aimed to assess the retention and caries-preventive effectiveness of a giomer-based pit and fissure sealant applied with and without bioactive glass air-abrasion pretreatment in young adults over a 12-month follow-up period. The null hypothesis of this study was that air-abrasion pretreatment using bioactive glass would not significantly affect the clinical performance of a giomer-based pit and fissure sealant in terms of retention and caries prevention compared with sealant application without pretreatment at 6 and 12 months.

## Participants and methods

The materials used in the present trial are summarized in Table 1 and were applied strictly in accordance with the manufacturers’ instructions. All operative procedures were performed by a single experienced clinician with over 13 years of clinical practice in restorative dentistry. The operator was not involved in outcome assessments or statistical analysis.

**Table 1:** Materials’ product name, manufacturer, description, composition, and lot number.

Product name	Manufacturer	Description	Composition	Lot number
Sylc Bioglass	Velopex International, London, UK	Bioactive glass air-abrasion powder for surface cleaning and enamel conditioning	Calcium sodium phosphosilicate bioactive glass (45S5-type); Elemental component: Silicon 21 wt%, Calcium 18 wt%, Sodium 18 wt%, Phosphorus 3 wt%, Oxygen 40 wt%)	10161
BeautiSealant Primer	Shofu Inc., Kyoto, Japan	Self-etching primer	Acetone, Purified water, Phosphonate monomer, Others	122281
BeautiSealant Paste	Shofu Inc., Kyoto, Japan	Giomer-based pit and fissure sealant	Dimethyl Aminoethyl Methacrylate 0.1-2 wt%, Glass powder 30–40 wt%, UDMA 25–30 wt%, TEGDMA, SiO2, Others	122297

### Ethical approval and trial registration

This study was designed, conducted, and reported in compliance with the Consolidated Standards of Reporting Trials (CONSORT) 2010 statement. The trial was prospectively registered in the ClinicalTrials.gov database (NCT06003452), with initial registration completed on 15 August 2023. The complete study protocol and statistical analysis plan can be obtained from the corresponding author upon reasonable request. All research procedures involving human participants were performed in accordance with the ethical standards outlined in the Declaration of Helsinki (2013 revision). Approval to conduct the study was granted by the Research Ethics Committee of the Faculty of Dentistry, Cairo University, and was obtained prior to the initiation of participant recruitment (approval ID: 32424). Written informed consent was secured from all participants before their inclusion in the study.

### Sample size calculation

The sample size was determined based on a previously published study [14], which reported a complete retention rate of 34.5% for fissure sealants applied without air-abrasion pretreatment. A two-tailed Z test for comparison of two independent proportions was used for the calculation, with the alpha level set at 5% and the statistical power at 80%. Based on these parameters, a minimum of 43 teeth per group was required to detect an absolute difference of 30% between the study groups. To account for potential attrition during the follow-up period, the calculated sample size was increased by 10%, resulting in a final sample of 48 teeth per group (96 teeth total), with one eligible molar contributed by each participant. Sample size estimation was performed using G*Power software (version 3.1.9.2; Heinrich Heine University Düsseldorf, Germany).

### Study design and setting

This investigation was designed as a randomized, double-blind, parallel-group clinical trial with a 1:1 allocation ratio and a superiority framework. The study was conducted at the Conservative Dentistry Clinics, Faculty of Dentistry, Cairo University, Egypt. Participant recruitment was conducted between June and August 2024. Following enrolment, interventions were delivered beginning in September 2024 and were completed over the subsequent weeks. Each Participant was followed for 12 months after sealant placement. The final follow-up assessments were completed in November 2025, marking the end of the overall study period. Participants were screened for eligibility according to predefined inclusion and exclusion criteria. Eligible individuals received comprehensive information regarding the study objectives, procedures, number of visits, anticipated benefits, and potential risks. Written informed consent was obtained from all participants prior to enrollment. Participants received standardized oral hygiene instructions, including the use of a soft-bristled toothbrush, twice-daily tooth brushing, and the modified Bass brushing technique. These instructions were reviewed and reinforced at each follow-up visit to ensure adherence.

Participants were randomly assigned to one of two study groups according to the allocated intervention. Clinical evaluation of the sealants was performed at baseline, 6 months, and 12 months. Sealant retention was assessed using Simonsen’s criteria, while the presence of secondary caries was evaluated using the modified United States Public Health Service (USPHS) criteria. No important amendments to the study protocol or predefined outcomes were made following trial initiation.

### Eligibility criteria

Participants were eligible if they were young adults aged 18–25 years who presented with fully erupted permanent mandibular first or second molars of normal anatomical form and retentive occlusal fissures classified as ICDAS codes 0 or 1. Eligible participants demonstrated good oral hygiene (plaque index score of 0 or 1), normal occlusion, no evidence of parafunctional occlusal activity, and willingness to participate in the study. Individuals were excluded if they had medical conditions potentially influencing oral health or study participation, experienced xerostomia, or presented with teeth that were non-vital, structurally compromised, or associated with defective adjacent or opposing restorations. Additional exclusion criteria included developmental enamel defects, periodontal involvement of the study tooth, and dentin hypersensitivity. To ensure independence of observations, only one eligible permanent molar was selected from each participant and included in the study.

### Randomization, sequence generation, and allocation concealment

Simple randomization was used to allocate participants in a 1:1 ratio. The random sequence was generated by an independent study member who was not involved in participant recruitment or clinical procedures. A list of random numbers ranging from 1 to 96 was created using an online random sequence generator (www.random.org), and group assignments were distributed equally between the intervention and control arms. Allocation concealment was ensured by placing the sequentially numbered assignments into sealed, opaque envelopes prepared by a contributor with no clinical involvement in the trial. At the time of the clinical procedure, each participant and his/her corresponding tooth were assigned to a study group by selecting one envelope. Due to the differences in application protocols between the study groups, blinding of the operator was not feasible. However, participants, outcome assessors, and the statistician were blinded to group allocation throughout the study.

### Operative procedures

**Fig. 1:**
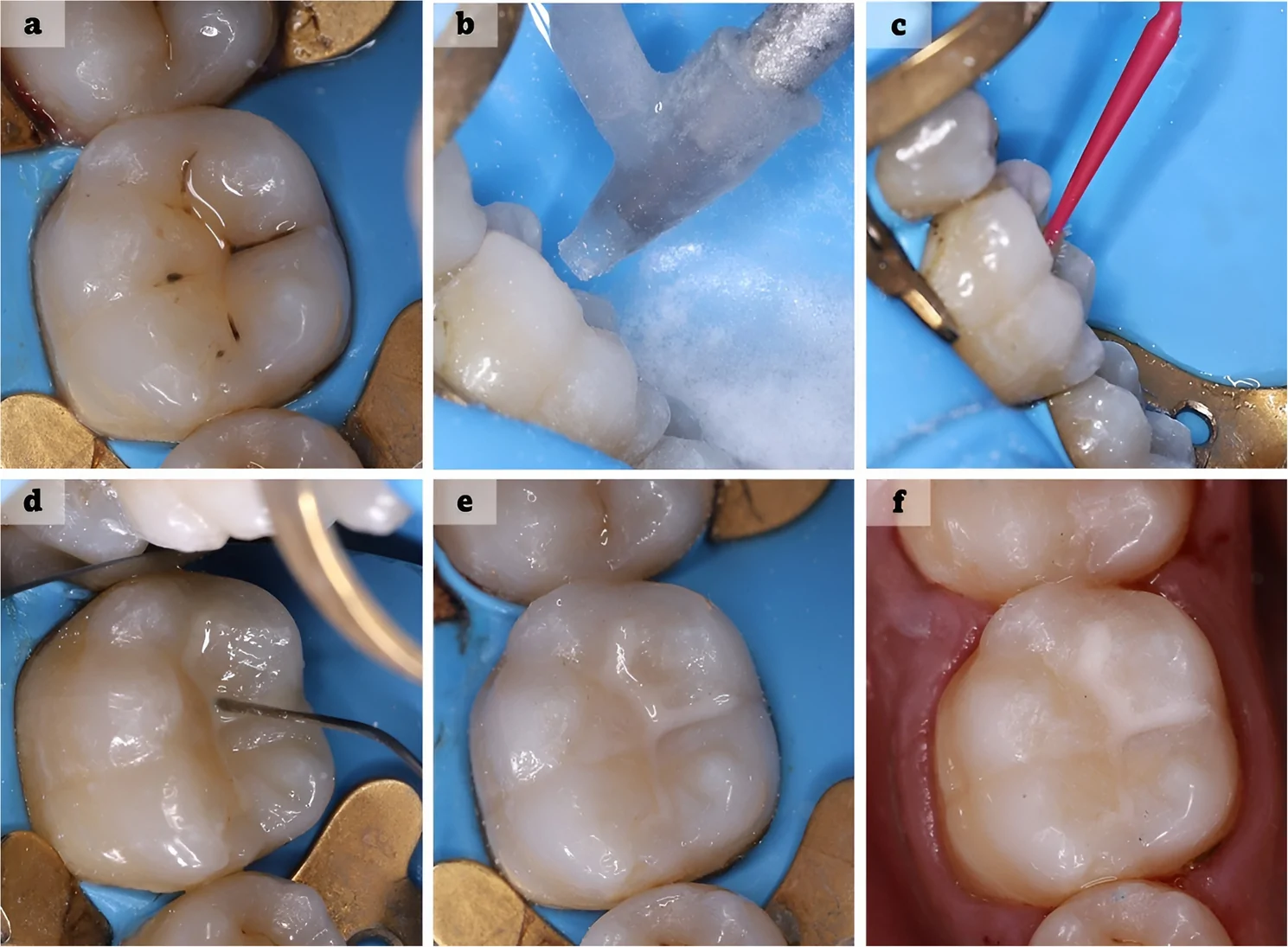
Clinical steps of sealant application on a representative tooth: (**a**) Preoperative view under rubber dam isolation; (**b**) Enamel surface conditioning using bioactive glass air-abrasion (intervention group only); (**c**) Application of self-etch primer; (**d**) Application of the giomer-based fissure sealant into the occlusal fissures; (**e**) Post-application view following light curing; (**f**) Immediate postoperative view after finishing and polishing

All procedures were performed by a single calibrated operator under standardized clinical conditions (Fig. 1). The selected tooth was isolated using a rubber dam (Sanctuary Dental Dam, Sanctuary Health Sdn Bhd, Perak, Malaysia) secured with an appropriately sized clamp (KSK Clamps, Dentech Corporation, Tokyo, Japan). Prior to sealant placement, the occlusal surface was cleaned to remove plaque and debris using a non-fluoridated prophylaxis paste (Prophy Paste, PSP Dental Co. Ltd., Kent, UK) applied with a low-speed prophylaxis brush (Nylon LATCH R.A. Prophylaxis Polishing Brushes, Dentamerica Inc., California, USA), followed by thorough rinsing with water and gentle air drying.

#### Intervention group (bioactive glass air-abrasion pretreatment)

In the intervention group, enamel pretreatment was performed using bioactive glass air-abrasion according to the manufacturer’s instructions. The occlusal surface was treated using an air-abrasion device (AquaCare™, Velopex International, London, UK) with bioactive glass powder (Sylc Bioglass, Velopex International, London, UK), which delivers the powder in a pressurized air-water stream. The device was operated at 60 psi particle energy and 5 g/min jet intensity for 20 s, using a working distance of approximately 10 mm and an impingement angle of 90° [15, 16]. Following air-abrasion, the treated surface was thoroughly rinsed with water and gently air-dried before sealant application.

After ensuring adequate dryness of the enamel surface, a self-etch primer (BeautiSealant Primer, Shofu Inc., Kyoto, Japan) was dispensed and actively applied to the pits and fissures using a disposable microbrush, left undisturbed for 5 s, and gently air-dried. A stronger air stream was subsequently applied until a thin, uniform bonding layer was obtained.

The sealant (BeautiSealant, Shofu Inc., Kyoto, Japan) was then injected directly into the pits and fissures using the manufacturer’s dispensing tip. The material was applied in a single direction to minimize air entrapment and ensure complete fissure penetration. Light polymerization was performed for 20 s, according to the manufacturer’s recommendations, using an LED light-curing unit (CuringPen, Changzhou Sifary Medical Technology Co., Jiangsu, China) operating at an irradiance of 1200 mW/cm^2^. Following curing, the sealed surfaces were examined with a dental explorer to verify complete coverage and absence of voids. Occlusion was checked using articulating paper and adjusted, when necessary, followed by finishing and polishing (OneGloss, Shofu Dental Corporation, Kyoto, Japan).

#### Comparator group (no pretreatment)

In the comparator group, no enamel pretreatment was performed. The occlusal surface was cleaned using the same prophylaxis protocol described above. After cleaning and drying, the self-etch primer and giomer-based sealant were applied according to the same protocol described for the intervention group.

### Outcome assessment

Sealant retention at 12 months was considered the primary outcome measure, whereas secondary caries incidence was assessed as a secondary outcome. Clinical evaluations were performed at baseline, 6 months, and 12 months following sealant placement. Outcomes were assessed by visual-tactile examination using a dental mirror and explorer under adequate illumination. Sealant retention was evaluated according to Simonsen’s criteria and classified as complete retention (sealant fully covering the pits and fissures), partial loss (partial loss of sealant with exposure of part of the fissure system), or total loss (complete absence of the sealant from the occlusal surface) [17]. Secondary caries was evaluated according to the modified USPHS criteria. Teeth exhibiting no evidence of discoloration, cavitation, or softened tooth structure adjacent to the sealant margins were classified as Alpha, whereas teeth showing evidence of secondary caries were classified as Charlie [18].

All clinical evaluations were performed independently by two examiners with more than 15 years of clinical experience in restorative dentistry. The examiners were not involved in the clinical procedures and were blinded to group allocation. Prior to study initiation, they underwent training and calibration in the application of Simonsen’s criteria and the modified USPHS criteria. Calibration was conducted through independent examination of teeth not included in the study sample. Inter-examiner agreement was excellent (Cohen’s κ = 0.82), and any disagreements during the study were resolved by consensus.

Participants were monitored throughout the follow-up period for any adverse events, complications, or unintended effects related to the interventions. The outcome measures and their corresponding assessment criteria are summarized in Table 2.

**Table 2:** Outcome measures and their corresponding clinical assessment criteria used for sealant evaluation during the follow-up period

Outcome category	Outcome	Method of assessment	Assessment criteria
Primary outcome	Sealant retention	Simonsen’s criteria	Complete retention (CR): Sealant fully intact on occlusal surface.
			Partial loss (PL): Sealant partially missing, with exposure of pits/fissures.
			Total loss (TL): Sealant entirely absent from occlusal surface.
Secondary outcome	Secondary caries	Modified USPHS criteria	Alpha (A): No evidence of caries.
			Charlie (C): Presence of caries.

### Statistical analysis

Statistical analysis was performed using MedCalc software (version 22.0; MedCalc Software Ltd., Ostend, Belgium). Categorical variables were summarized as frequencies and percentages. Intergroup comparisons were conducted using the Chi-square test. Intragroup comparisons across the different follow-up intervals were analyzed using Cochran’s Q test, followed by multiple comparisons. The level of statistical significance was set at *P* ≤ 0.05 for all analyses. Relative risk was calculated to assess clinical significance. All statistical tests were two-tailed, and results were reported with a 95% confidence interval. The statistical power was set at 80%. Because only one tooth was included per participant, the tooth and participant represented the same unit of analysis. All randomized participants completed the study; therefore, no missing data-handling was required.

## Results

### Participant flow and baseline characteristics

**Fig. 2:**
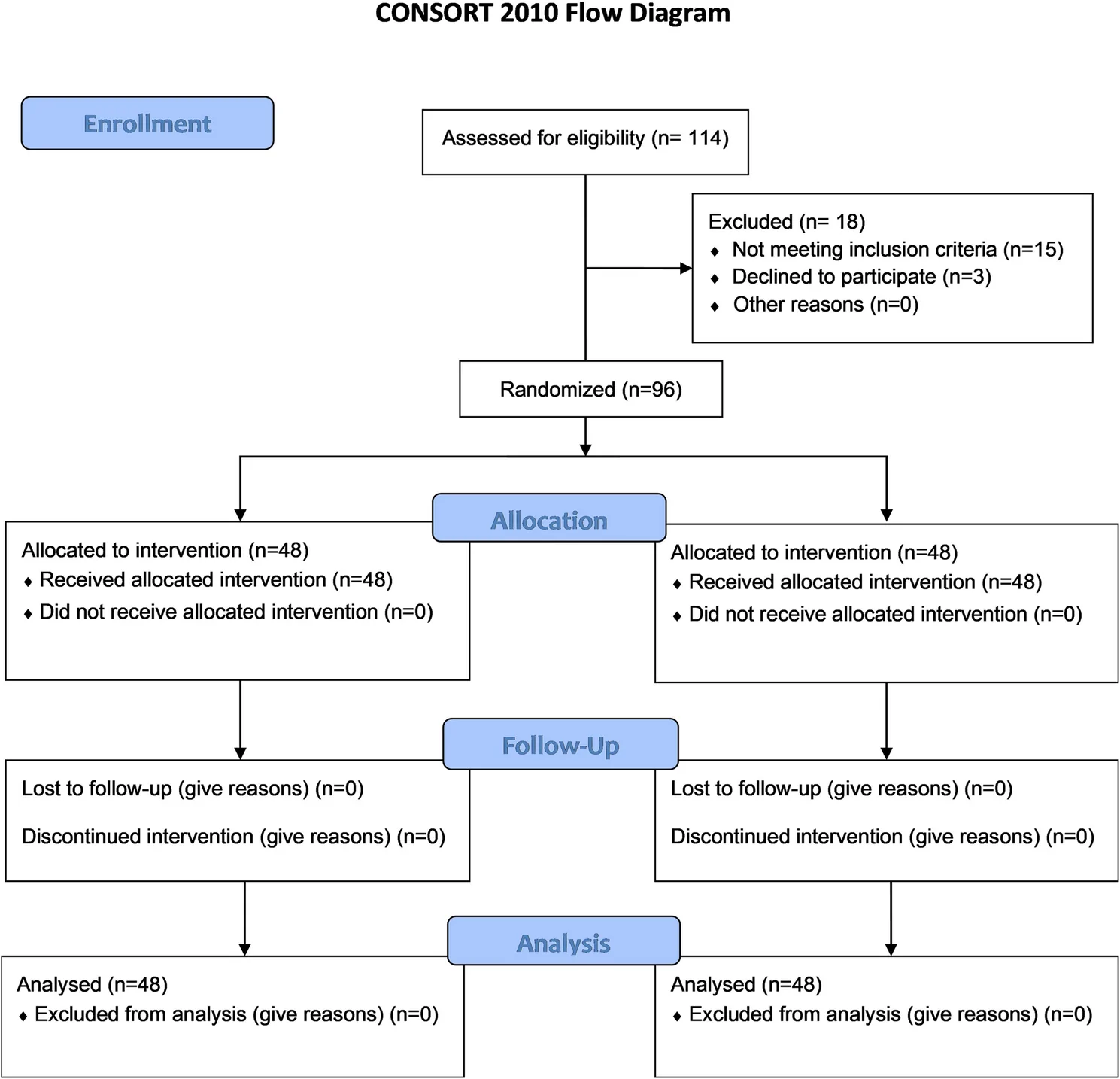
CONSORT Flow Diagram

A total of 96 participants, each contributing one eligible permanent molar, were included in the study. Accordingly, 96 teeth were randomly allocated to either the intervention group or the comparator group, with 48 teeth assigned to each group. Complete follow-up was achieved for all participants, and all sealed teeth were available for clinical evaluation at baseline, 6 months, and 12 months (Fig. 2). The study population consisted of 47 males (49%) and 49 females (51%), with a mean age of 20.17 ± 2.03 years. No statistically significant differences were observed between the two groups with respect to age (*P* = 0.952), gender distribution (*P* = 0.683), or tooth type distribution (*P* = 0.409), indicating comparable baseline characteristics (Table 3). No adverse events, complications, or unintended effects related to either intervention were reported during the study period.

**Table 3:** Baseline demographic and clinical characteristics of participants in both study groups. Age is presented as mean ± standard deviation, whereas gender and tooth type are presented as frequency and percentage

Variable	Category	Giomer-based Sealant with BAG air-abrasion pretreatment (Intervention group)	Giomer-based Sealant without pretreatment (Comparator group)	*P* value
Age	Years	20.12 ± 1.97	20.21 ± 2.10	0.829
Gender	Female	23 (47.9%)	26 (54.2%)	0.683
	Male	25 (52.1%)	22 (45.8%)	
Tooth type	Mandibular first molar	18 (37.5%)	23 (47.9%)	0.409
	Mandibular second molar	30 (62.5%)	25 (52.1%)	

### Sealant retention

At baseline, all teeth in both groups demonstrated complete sealant retention, with no statistically significant difference between groups (*P* = 1.000). After 6 months, complete retention was observed in 91.7% of teeth in the intervention group and 75% in the comparator group; however, the difference between groups was not statistically significant (*P* = 0.068). At the 12-month follow-up, a statistically significant difference in sealant retention was detected between the two groups (*P* < 0.0001). The intervention group demonstrated a higher percentage of complete retention (87.5%) compared with the comparator group (33.3%). Partial loss was observed in 10.4% and 54.2% of teeth in the intervention and comparator groups, respectively, while total loss occurred in 2.1% of teeth in the intervention group and 12.5% in the comparator group (Table 4 ).

**Fig. 3:**
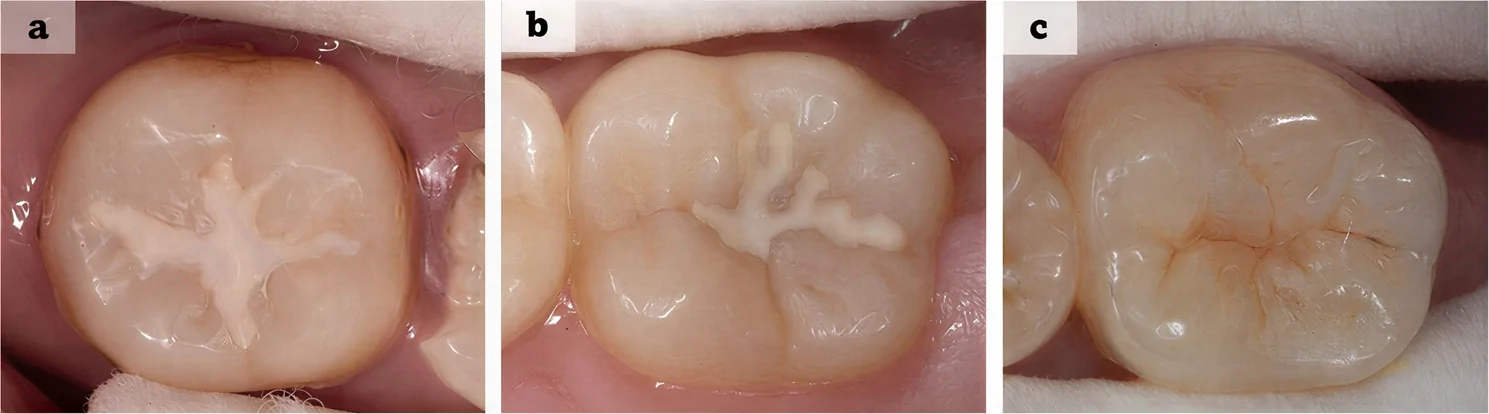
Representative clinical photographs illustrating sealant retention at 12-month follow-up: (**a**) Complete retention; (**b**) Partial loss; (**c**) Total loss

Intragroup analysis revealed a statistically significant change in retention scores over time within both groups (intervention group: *P* = 0.009; comparator group: *P* < 0.001). Relative risk analysis showed that teeth in the intervention group exhibited an 81.25% lower risk of sealant retention failure compared with the comparator group at 12 months (RR = 0.1875; 95% CI: 0.0864–0.4069; *P* < 0.0001)]. Representative clinical photographs illustrating complete retention, partial loss, and total loss of the fissure sealant at follow-up are presented in Fig. 3.

**Table 4:** Frequency and percentage distribution of sealant retention scores assessed using Simonsen's criteria at baseline, 6 months, and 12 months for both study groups

Follow-up	Giomer-based Sealant with BAG air-abrasion pretreatment (Intervention group)				Giomer-based Sealant without pretreatment (Comparator group)				*P* value
	Rank	Complete retention	Partial loss	Total loss	Rank	Complete retention	Partial loss	Total loss	
Baseline	A	48 (100%)	0 (0%)	0 (0%)	A	48 (100%)	0 (0%)	0 (0%)	*P*=1.0000
6 months	AB	44 (91.7%)	4 (8.3%)	0 (0%)	B	36 (75%)	10 (20.8%)	2 (4.2%)	*P*=0.0682
12 months	B	42 (87.5%)	5 (10.4%)	1 (2.1%)	C	16 (33.3%)	26 (54.2%)	6 (12.5%)	*P*<0.0001*
*P* value	*P*=0.009*				*P*<0.001*				

*Statistically significant difference (*P* ≤ 0.05)

### Secondary caries incidence

No secondary caries lesions were detected in either group at baseline, 6 months, or 12 months (Table 5 ). Intergroup comparisons revealed no statistically significant differences between the intervention and comparator groups at any evaluation time point (*P* = 1.000). Intragroup analysis showed no statistically significant changes in secondary caries incidence over time within either group (*P* = 1.000). Relative risk analysis demonstrated no difference in caries occurrence between groups at the 12-month follow-up (RR = 1.0000; 95% CI: 0.02024–49.3999; *P* = 1.0000).

**Table 5:** Frequency and percentage distribution of secondary caries scores assessed using the modified USPHS criteria at baseline, 6 months, and 12 months for both study groups

Follow-up	Giomer-based Sealant with air-BAG abrasion pretreatment (Intervention group)		Giomer-based Sealant without pretreatment (Comparator group)			*P* value
	Alpha score	Charlie score	Alpha score		Charlie score	
Baseline	48 (100%)	0 (0%)		48 (100%)	0 (0%)	*P* = 1.0000
6 months	48 (100%)	0 (0%)		48 (100%)	0 (0%)	*P* = 1.0000
12 months	48 (100%)	0 (0%)		48 (100%)	0 (0%)	*P* = 1.0000
*P* value	*P* = 1.0000			*P* = 1.0000		

## Discussion

The present randomized clinical trial evaluated the effect of bioactive glass (BAG) air-abrasion pretreatment on the clinical performance of a giomer-based fissure sealant in young adults over a 12-month period, with specific emphasis on sealant retention and secondary caries prevention. The null hypothesis stated that no differences would exist between sealants placed with or without BAG air-abrasion pretreatment in terms of retention or caries preventive efficacy.

Based on the study findings, the null hypothesis regarding sealant retention was rejected. Giomer-based sealants applied following BAG air-abrasion demonstrated significantly superior retention at 12 months compared with sealants placed without pretreatment, with the intervention group showing an 81.25% lower risk of retention failure. In contrast, no differences in secondary caries incidence were detected between groups during the follow-up period.

During early follow-up intervals, retention outcomes were comparable between groups; however, statistically significant differences emerged after 12 months. These findings suggest that enamel surface conditioning may play an important role in enhancing the long-term bonding effectiveness of self-etch sealant systems. This interpretation is consistent with previous research emphasizing the importance of adequate enamel preparation for durable sealant retention. Several studies have demonstrated that BAG air-abrasion enhances enamel surface reactivity, improves etchability, and supports adhesive performance comparable to that achieved with conventional alumina air-abrasion techniques [10, 19, 20].

Conversely, other studies have reported lower sealant retention following BAG air-abrasion when compared with conventional alumina-based abrasion. This discrepancy has largely been attributed to the distinct physical characteristics of BAG particles, including their relatively lower hardness and rounded particle morphology, which result in a milder enamel surface modification and a less pronounced micromechanical retentive pattern [21]. Unlike aluminum oxide, which produces aggressive mechanical cutting of enamel, BAG particles are not primarily designed for enamel removal. Although in vitro investigations have demonstrated that BAG powders can abrade the enamel surface, this process occurs at a slower rate and with reduced efficiency compared to alumina, supporting the view that their primary function is surface cleaning and polishing rather than aggressive enamel roughening [22, 23].

Taken together, the superior retention outcomes observed in the present study may be attributed primarily to improved surface conditioning achieved by BAG air-abrasion pretreatment. By improving fissure cleanliness, removing organic debris, and producing mild enamel surface modification, BAG air-abrasion may enhance the quality of the bonding substrate before sealant placement [19, 21]. This effect may be particularly important when used in conjunction with a self-etch primer, whose limited etching capacity can benefit from a cleaner and more receptive enamel surface [10, 20, 21].

Although the addition of BAG air-abrasion significantly improved sealant retention, the comparatively poor performance of the self-etch-only group warrants attention. These findings suggest that the cleansing and etching capacity of the self-etch primer alone may be insufficient to achieve durable micromechanical retention on unprepared enamel surfaces. Several clinical studies have similarly reported limited longevity and increased early failure rates of giomer-based sealants when applied without prior enamel pretreatment [24, 25, 26]. This reduced performance has been attributed to the weak demineralizing effect of mild self-etch primers, which are less effective than phosphoric acid etching in removing the aprismatic enamel layer [26]. In addition, mechanical cleaning with a prophylaxis brush alone may be insufficient to eliminate organic debris lodged within deep and narrow fissures [24]. Because self-etch primers lack a separate rinsing step, residual contaminants may remain at the base of the fissures, thereby compromising resin penetration and weakening the adhesive interface [24, 25]. 

The findings of the present study suggest that while acceptable early retention may be achieved without pretreatment, adjunctive enamel surface conditioning plays an important role in enhancing the long-term durability of giomer-based sealants. In agreement with this interpretation, a recent systematic review has demonstrated that combined mechanical pretreatment protocols provide superior sealant retention over acid etching alone, particularly over extended follow-up periods [27].

With respect to secondary caries, no lesions were detected in either group during the 12-month follow-up period. These findings align with previous reports demonstrating that sealants may continue to provide caries protection even in cases of partial or complete retention loss, owing to the persistence of residual material and ion-releasing particles within fissure depths [28]. Similarly, regression or arrest of early carious lesions following the application of giomer-based sealants has been reported and attributed to their remineralization potential [26, 29]. Laboratory and clinical studies have suggested that the bioactivity of giomer materials may be attributed to the presence of surface pre-reacted glass ionomer (S-PRG) fillers, which are capable of releasing fluoride, borate, strontium, sodium, silicate, and aluminum ions. These ions may contribute to enhanced enamel acid resistance, buffering of acidic environments, promotion of remineralization, and inhibition of cariogenic bacterial activity [6, 30, 31]. Specifically, fluoride and strontium ions have been reported to promote the formation of more acid-resistant apatite structures, while borate, sodium, and strontium may contribute to pH buffering and remineralization processes [25, 31]. In addition, BAG particles have been reported to release calcium, phosphate, sodium, and silicate ions upon exposure to saliva, which may contribute to mineral deposition, enhancement of enamel acid resistance, and promotion of remineralization [12, 19, 22]. However, because no carious lesions developed during the follow-up period and remineralization was not directly assessed, the present study cannot determine the extent to which these bioactive effects influenced clinical outcomes. Collectively, these observations support the concept that giomer sealants may provide not only a physical barrier but also additional biofunctional benefits that could contribute to caries prevention [32].

Overall, the present randomized clinical trial adds clinical evidence supporting the use of bioactive glass air-abrasion pretreatment in conjunction with a self-etch giomer-based sealant to enhance sealant retention outcomes. These findings highlight the clinical importance of appropriate enamel surface conditioning in optimizing the durability of self-etch fissure sealant systems. Nevertheless, several limitations should be acknowledged when interpreting these results. First, the study was conducted at a single academic center, which may limit the external validity and generalizability of the findings to other clinical settings and populations. Second, the study population consisted exclusively of young adults aged 18–25 years with fully erupted permanent molars. Although contemporary clinical guidelines recommend fissure sealant application according to caries risk and fissure morphology rather than age alone, these findings should be interpreted with caution when extrapolated to younger children, older adults, or individuals with different caries risk profiles and enamel characteristics. Third, the follow-up period was limited to 12 months. Although this duration was sufficient to identify significant differences in sealant retention, it may not adequately capture long-term retention behavior or secondary caries development. The absence of secondary caries lesions in either group may be attributable to the relatively short follow-up period and the low incidence of caries within the study population. In addition, the present study evaluated bioactive glass air-abrasion pretreatment in conjunction with the manufacturer’s recommended self-etch protocol. Other enamel conditioning approaches, including conventional phosphoric acid etching, were not evaluated and therefore warrant investigation in future studies. Furthermore, all clinical procedures were performed by a single operator. Although this approach enhanced procedural standardization and minimized inter-operator variability, the findings should be confirmed in multi-operator settings. Finally, simple randomization rather than block or stratified randomization was used. Although baseline characteristics were well balanced between groups, alternative allocation methods may provide additional assurance of group comparability in future studies.

Future research should include larger sample sizes, extended follow-up periods beyond 12 months, and assessment across different enamel substrates, such as molar incisor hypomineralization (MIH). Additionally, incorporating regular clinical monitoring and timely resealing into preventive care protocols is essential to maintain continuous fissure protection.

## Conclusions

Bioactive glass air-abrasion pretreatment significantly enhanced the retention of giomer-based fissure sealant compared with non-abraded application, while no differences in secondary caries incidence were detected between groups over the 12-month evaluation period. These findings support the clinical effectiveness of giomer-based sealants for fissure protection and emphasize the importance of enamel surface conditioning in optimizing long-term retention.

## Data Availability

The datasets generated and/or analyzed during the current study are available from the corresponding author upon reasonable request.
